# Surgical suture-assisted endoscopic band ligation for the management of recurrent hemostasis after unsuccessful use of hemoclips for diverticular bleeding

**DOI:** 10.1055/a-2183-6154

**Published:** 2023-11-20

**Authors:** Shaoxiong Zeng, Qu Zhang, Qin Li, Qingtian Luo, Minwen Jiang, Chun-sheng Cheng

**Affiliations:** 1 Department of Gastroenterology and Endoscopy Center, Huazhong University of Science and Technology Union Shenzhen Hospital (Nanshan Hospital) and the 6th Affiliated Hospital of Shenzhen University Health Science Center, Shenzhen, China

A 47-year-old obese man presented with acute-onset lower abdominal pain and hematochezia of unknown etiology, accompanied by dizziness and fatigue. His stool was characterized as dark red and watery, and of significant volume. The patient underwent an emergency colonoscopy, during which intermittent hemorrhage was observed at the site of a transverse colon diverticulum ( Fig. 1 **a** ). Three hemostatic clips were successfully placed to occlude the diverticulum during the endoscopic procedure, and bleeding was observed to have ceased.

**Fig. 1:**
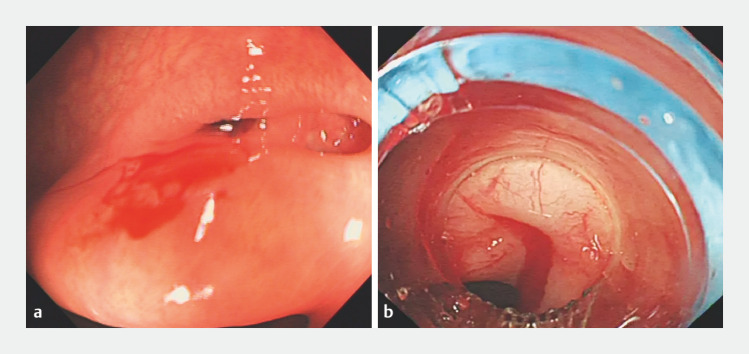
Endoscopic images showing: **a** hemorrhage at the site of a transverse colon diverticulum; **b** 30 days later, recurrent active bleeding at the specific location of the transverse colon diverticulum.

Unfortunately, after a period of 30 days, the patient experienced another episode characterized by significant amounts of dark red bloody stools. Subsequently, a second emergency colonoscopy was conducted, which revealed active bleeding at the specific location of the transverse colon diverticulum ( Fig. 1 **b** ). Owing to the failure of hemostasis with hemoclips, endoscopic band ligation was attempted as an alternative. However, owing to a short trigger wire of the ligator, it was not possible to attach the band ligation device to the colonoscope. To overcome this challenge, we innovatively used surgical suture to extend the trigger wire of the ligator, thereby enabling successful connection of the device to the tip of the colonoscope ( Fig. 2 ). The diverticulum in the transverse colon was suctioned into the cap of the endoscopic ligator, and the elastic O ring was subsequently released. Following repeated irrigation, no further bleeding was observed ( Fig. 3 ; Video 1 ). During follow-up for 3 months, there was no recurrence of bleeding, and no perforation or abscess formation.

**Fig. 2:**
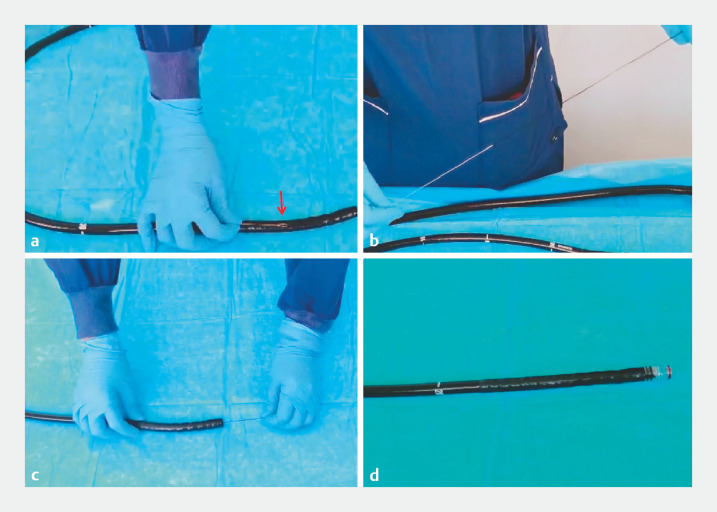
Photographs showing: **a** that the length of the trigger wire in the ligation device (red arrow) was shorter than the length of the colonoscope; **b,c** the trigger line being extended with surgical suture; **d** the ligator successfully installed on the colonoscope.

**Fig. 3:**
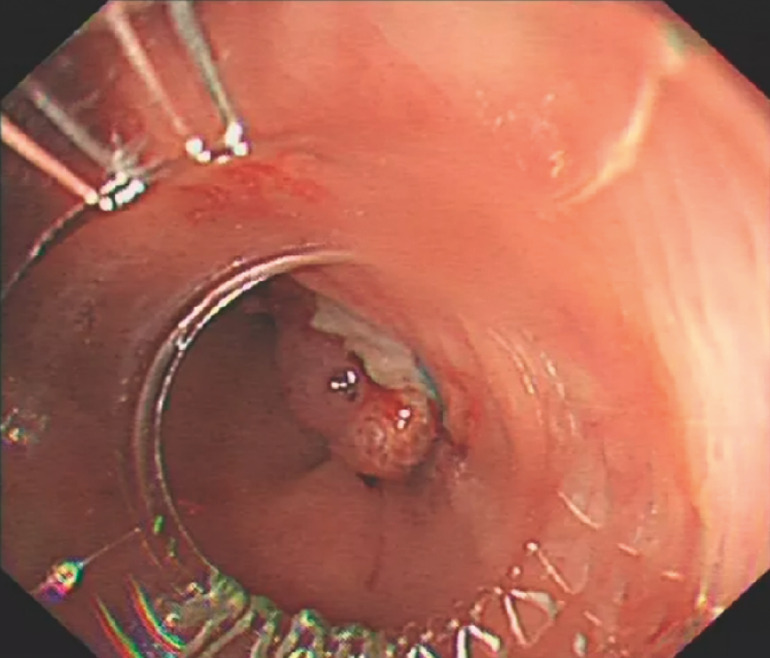
Endoscopic image showing no evidence of further bleeding after the surgical suture-assisted endoscopic band ligation procedure.

Surgical suture-assisted endoscopic band ligation is performed to manage a case of recurrent diverticular bleeding after unsuccessful hemostasis with hemoclips.Video 1

The treatment modalities for colonic diverticular bleeding encompass endoscopic clipping, endoscopic band ligation, and injection therapy 1 . Conventional endoscopic clipping and local epinephrine injection methods may not always be effective in the prevention or treatment of rebleeding 2 . The use of surgical suture to elongate the trigger wire of the ligator can facilitate successful installation of the band ligation device onto the colonoscope to manage colonic diverticular bleeding, resulting in immediate hemostasis. This can serve as a secure and effective rescue approach, particularly in patients who have experienced unsuccessful hemostasis with clips. This technique is worthy of clinical promotion.

Endoscopy_UCTN_Code_TTT_1AQ_2AZ

## Notes

Endoscopy E-Videos https://eref.thieme.de/e-videos
*E-Videos* is an open access online section of the journal *Endoscopy* , reporting on interesting cases and new techniques in gastroenterological endoscopy. All papers include a high-quality video and are published with a Creative Commons CC-BY license. Endoscopy E-Videos qualify for HINARI discounts and waivers and eligibility is automatically checked during the submission process. We grant 100% waivers to articles whose corresponding authors are based in Group A countries and 50% waivers to those who are based in Group B countries as classified by Research4Life (see: https://www.research4life.org/access/eligibility/ ). This section has its own submission website at https://mc.manuscriptcentral.com/e-videos .
